# Intraoperative magnetic resonance imaging during surgical resection for drug resistant epilepsy eliminates the incidence of inadvertent incomplete resection of the epileptogenic zone and early surgical failure

**DOI:** 10.1007/s00381-025-06925-y

**Published:** 2025-09-11

**Authors:** Joyce Koueik, Youngwon Youn, Susan Rebsamen, Adam N. Wallace, Adam Kney, Andrew T. Knox, David A. Hsu, Raheel Ahmed

**Affiliations:** 1 Department of Neurological Surgery, School of Medicine and Public Health, University of Wisconsin, 1675 Highland Ave, Madison, WI 53792-3252 USA; 2 Department of Radiology, School of Medicine and Public Health, University of Wisconsin, Madison, WI USA; 3 Department of Neurology, School of Medicine and Public Health, University of Wisconsin, Madison, WI USA

**Keywords:** Epileptogenic zone, Intraoperative magnetic resonance imaging, Seizure freedom

## Abstract

**Objective:**

We present a case series of pediatric subjects with drug resistant epilepsy (DRE) that underwent surgical resection utilizing intraoperative MRI (IOMRI) to confirm resection of the hypothesized epileptogenic zone (EZ). Clinical, radiographic and seizure control outcome measures are presented to support the hypothesis that inclusion of IOMRI enables assessment and confirmation of total resection of the hypothesized EZ and eliminates the incidence of inadvertent incomplete resections.

**Methods:**

We reviewed records for all pediatric patients (< 21 years) with DRE who underwent surgical resection between Dec 2017 and Aug 2023.

**Results:**

Thirty subjects with a mean follow-up duration of 4.2 ± 0.3 years (range: 1.6–7.3 years) were identified. The most prevalent pathological subtypes were focal cortical dysplasia (33%) followed by gliosis (30%). Phase II evaluation was undertaken in 24 subjects (80%). IOMRI revealed incomplete resection of the intended EZ in 73% (*n* = 22) of subjects, who then underwent additional resection. Two subjects underwent an incomplete resection due to functional constraints. Seizure improvement (Engel I + II) following surgery was observed in 90% of subjects with 80% (*n* = 24) subjects with Engel I outcome. An incomplete resection due to functional overlap or widespread network was associated with Engel III outcome in 3 subjects (10%).

**Conclusions:**

Intraoperative imaging during surgical resection for DRE eliminates the incidence of inadvertent incomplete resections and reduces the risk of early seizure recurrence. Our results over an extended follow-up period, elucidate long-term epilepsy outcomes for DRE surgery with IOMRI assistance, that are associated with a low incidence of repeat resection due to epilepsy recurrence.

## Introduction

Long-term rates of seizure freedom following epilepsy surgery decline over time [1]. Incomplete resection is the most common reason for early seizure recurrence following surgery for drug-resistant epilepsy (DRE) [2–4]. For DRE surgical candidates, surgery is targeted at the hypothesized EZ, derived from phase I and II evaluations, that delineates a surgical resection volume that needs to be removed completely, while avoiding functional deficits, to achieve seizure freedom [5]. In lesional epilepsy, the epileptogenic zone (EZ) often comprises of an epileptogenic lesion at the epicenter, and a zone of extra-lesional tissue that includes a wider irritative and seizure propagation zone [5]. In non-lesional epilepsy, which comprises 50% of subjects [6], the risk of an incomplete resection is higher, given the absence of a radiographic marker to guide EZ localization.

Various surgical adjuncts have been utilized to facilitate resection of the hypothesized EZ. These adjuncts rely on concordant data and include intraoperative electrocorticography (iECOG) to verify removal of spike activity [7], functional neuronavigation to identify eloquent cortex [8], and intraoperative imaging including ultrasound [9] and Magnetic Resonance Imaging (MRI) to confirm resection [10, 11]. Although these studies have demonstrated improved treatment outcomes with IOMRI-assisted surgeries, they are limited by their scope of application [12, 13], patient candidacy and availability [14]. Heterogeneity in application of these modalities underscores the importance of optimizing intraoperative assessment and confirmation of complete resection of the hypothesized EZ, to optimize the goal of seizure freedom.

Since long-term quality of life measures in children with DRE correlate with seizure control outcomes [15, 16], we aimed to investigate surgically modifiable risk factors for early surgical treatment failures. We focused on the role of intraoperative MRI (IOMRI) in facilitating complete and verifiable resection of the hypothesized EZ, and its impact on early seizure freedom. We hypothesized that complete surgical resection of the hypothesized EZ reduces the risk for early surgical failure and that IOMRI application facilitates complete resection of the EZ and impacts seizure control outcomes. We present a case series of pediatric DRE subjects that underwent IOMRI-assisted surgical resection. Clinical, radiographic and seizure outcome measures are presented to support our hypothesis by demonstrating that inclusion of IOMRI, enables assessment and confirmation of total resection of the hypothesized EZ, eliminates the risk of inadvertent incomplete resection, and is associated with durable seizure relief during early follow-up.

## Methods

We conducted a retrospective review of pediatric subjects with DRE who underwent surgical treatment for DRE with IOMRI assistance from Dec 2017 to Aug 2023. Inclusion criteria were as follows: (1) subjects with DRE (failure to control seizures despite adequate trial of two or more anti-seizure medication (ASM)), (2) age < 21 years at epilepsy surgery, and (3) post-surgical follow-up of at least 1 year. All consecutive patients that met the inclusion criteria were included over the study period. Exclusion criteria were as follows: (1) subjects who underwent hemispheric disconnections and (2) subjects with underlying (epileptogenic) tumors and tumor-related epilepsy. Demographic, clinical and surgical variables were analyzed. The study was approved by the University of Wisconsin, Madison Institutional Review Board.

### Surgical treatment and intra-operative MR imaging

All patients underwent Phase I evaluation, including clinical history with seizure semiology, neurologic examination, neuropsychology assessment, standard and high-density scalp EEG, structural and functional (in select cases) MRI, PET and SPECT studies as deemed necessary by the primary epileptologist. The Phase I – derived localization hypothesis was reviewed within a multi-disciplinary epilepsy conference. In the absence of concordant data, patients were recommended to undergo Phase II invasive stereo EEG (SEEG) monitoring, with attention to the onset and propagation of ictal and inter-ictal electrographic features. Intracranial EEG recordings of ictal- and inter-ictal events, electro-cortical stimulations and functional mapping data (where applicable) were reviewed within the epilepsy group. Subjects with a consensus, concordant localization hypothesis who were recommended to undergo surgical resection of the hypothesized EZ were included in the study.

The hypothesized EZ, based on Phase I and II evaluations, was used to formulate a surgical resection plan through anatomical-functional-electro-clinical correlations and multi-modal integration of diagnostic studies as described earlier [17]. The Medtronic Stealth Planning and Navigation software (S8; Minneapolis, MN) was used for preoperative planning and intraoperative neuronavigation respectively, for all cases. Preoperatively, the Medtronic planning software was used to co-register presurgical anatomical MRI and post-SEEG implantation CT studies. In select cases, presurgical fMRI and DTI studies were additionally co-registered and used to identify eloquent regions and reconstruct related white matter fiber tracts. A pre-operative 3-dimensional, volumetric surgical resection region was delineated, targeting the hypothesized EZ, that was then traced in 3-dimensional axial, sagittal and coronal views on presurgical anatomical T1-weighted post-contrast images. This volumetric, hypothesized EZ incorporated anatomical landmarks, SEEG electrode positions involved in seizure onset and early propagation and functional mapping data (as applicable), and was pre-surgically identified as the intended goal of surgical resection. This surgical plan was transferred to the Medtronic Navigation software for patient registration and intraoperative guidance during surgery.

During surgery, resection of the hypothesized EZ was undertaken based on anatomical landmarks, functional mapping/monitoring (as applicable) and intraoperative electrocorticography in select cases. Resection was continued till deemed complete based on surgical assessment and/or verification through neuronavigation. Surgical resection was halted if surgical landmarks precluded definitive confirmation for completeness of resection. At this stage, a temporary surgical closure was performed, and following a standardized safety check, the patient was transferred to the adjoining MRI suite. All subjects underwent the IOMRI study on the same scanner (1.5-T; 450 s Wide Bore, GE Medical Systems) that included standardized imaging sequences including: (1) volumetric T1-weighted axial sequences (TE 4.5 ms, TR 10.9 ms, matrix 320 X 320, slice thickness 1.5 mm, FOV 240 mm); (2) volumetric T2-weighted Fluid Attenuation Inversion Recovery weighted-CUBE (GE) sequences (TE max, TR 6000 ms, matrix 256 X 224, slice thickness 1.6 mm, FOV 250 mm), and (3) Diffusion-weighted sequences (TE 1, TR 9000 ms, matrix 128 X 256, slice thickness 5 mm, FOV 260 mm). Volumetric acquisitions were reconstructed in similar 3-plane orientation. Optional sequences included volumetric T2 CUBE non-gated sequences, and/or a post-contrast volumetric T1 weighted sequence when needed.

The IOMRI study was co-registered to the preoperative reference study and was reviewed conjointly with a neuroradiologist. The IOMRI study was interpreted focusing on volumetric comparison between the a priori, pre-surgical resection plan comprising of the hypothesized EZ and the actual resection achieved. A complete surgical resection was identified if the hypothesized EZ, intended as the goal of surgical resection, was entirely resected. If the IOMRI study identified any residual tissue within the hypothesized EZ, further resection was undertaken as guided by the IOMRI exam. In this event, the new volumetric IOMRI study was uploaded to the neuronavigation software and was used as the updated reference study with repeat patient registration. After completion of additional resection, a final standard, multilayered anatomical closure was undertaken. All subjects underwent a single intraoperative MRI study. All patients underwent an MRI study on post-operative day one with a similar image sequence protocol. The postoperative day 1 (POD 1) MRI study was similarly compared to the preoperative surgical resection plan to evaluate the extent of resection.

For anterior temporal lobectomies, the resection zone included mesial temporal structures, including uncus, amygdala extending superiorly to the carotid-choroidal line, complete hippocampectomy and parahippocampectomy extending posteriorly to the tectal plate, and complete resection of the lateral neocortical lobe including the superior temporal gyrus extending 4 cm on the dominant and 6 cm on the non-dominant lobe from the temporal pole respectively. For tailored, lesionectomy-plus resections, the hypothesized EZ encompassed the radiographic lesion with surrounding regions as supported by Phase II evaluation, and supplemented by ECOG when applicable. A motor function-sparing approach was used for frontal lobectomy [18], with resection extending posteriorly to the pre-central sulcus and including removal of the subcallosal region.

For data analysis and interpretation, completeness of resection of the hypothesized EZ was compared between the pre-, intra- and post-operative MRI studies respectively.

## Results

**Fig. 1:**
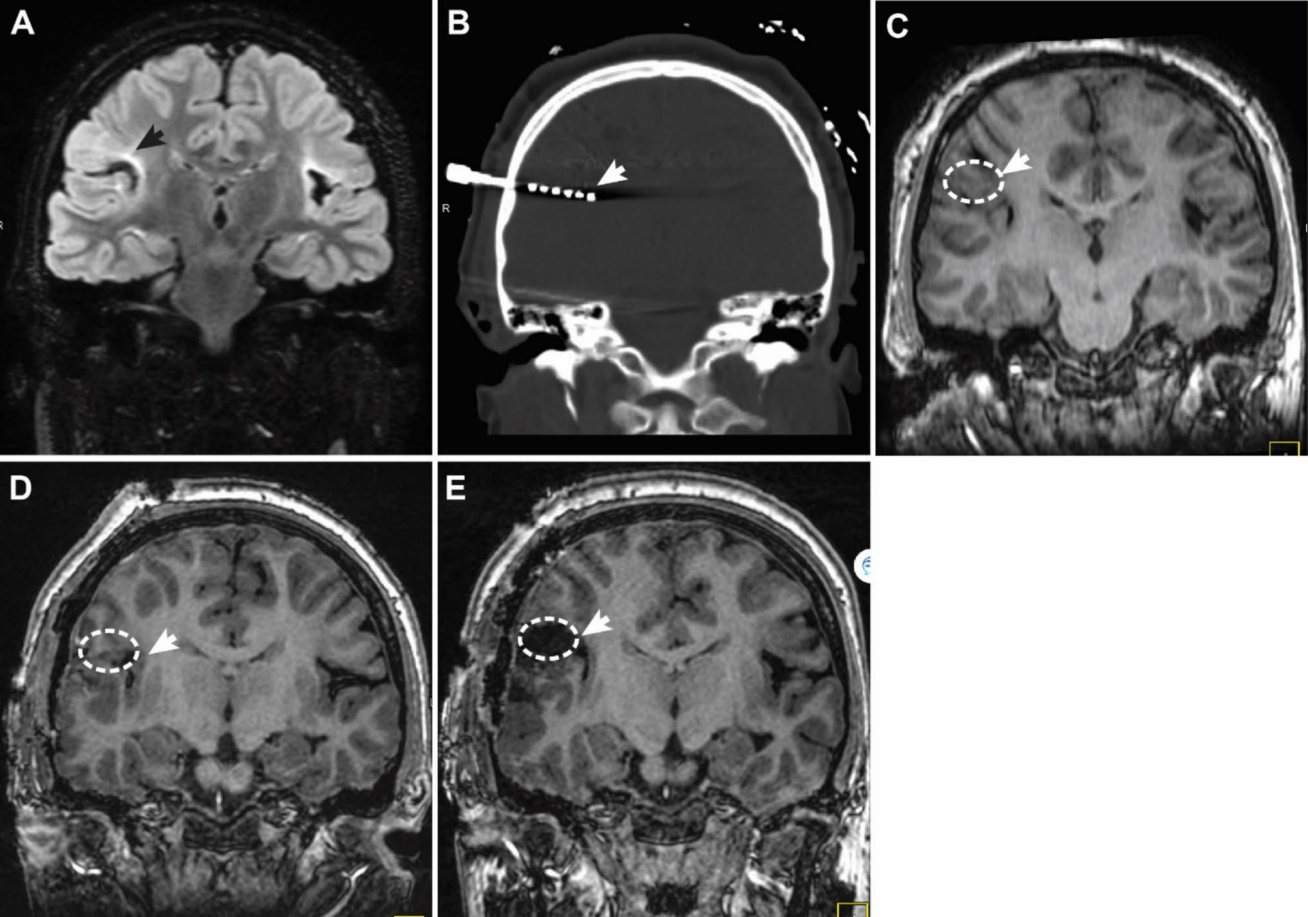
Illustrated example for Phase II guided EZ resection in a 10-year-old with speech and motor arrest. The patient had neonatal-onset hypoxic seizures and scalp EEG indicated frontal-opercular onset seizures. **A** Coronal T2-FLAIR weighted image indicating gliosis (black arrow) within posterior inferior frontal operculum. **B** Post-SEEG implantation CT image, coronal view, highlighting electrode placement within posterior frontal opercular cortex involved in seizure onset (white arrow) as evidenced by intracranial EEG recordings. **C** Presurgical coronal T1-weighted image with the EZ outlined (white dashed region) based on Phase II evaluation. After initial resection, patient experienced temporary seizure relief. Following seizure recurrence, imaging indicated **D** residual tissue (white dashed region) within intended EZ as highlighted on representative postsurgical coronal T1-weighted image. Repeat resection targeting the residual EZ yielded seizure relief. **E** Postsurgical coronal T1 weighted imaging after repeat surgery shows removal of the hypothesized EZ (white dashed region)

Our initial experience with EZ resection for ill-defined lesions like gliosis indicated that incomplete resection of the EZ was responsible for early seizure recurrence (Fig. 1). We henceforth decided to incorporate IOMRI in our surgical work flow for all epileptogenic lesion resections.

### Patient demographics

**Fig. 2:**
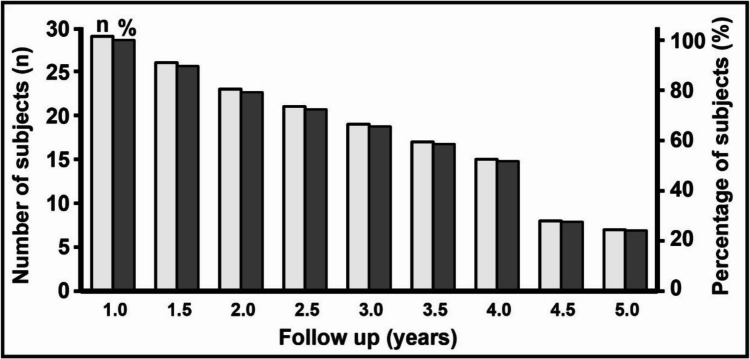
Graphical representation of postsurgical follow up for study cohort. All subjects had at least 1 year follow-up. Minimal follow-up was 1.1 years and 80% of subjects had at least a 2 year follow-up

Our cohort comprised of 30 subjects that underwent IOMRI-assisted surgical resection. Patient demographics are summarized in Table 1. The mean post-surgical follow-up for the cohort was 4.2 ± 0.3 years (range: 1.6–7.3 years; median: 4.6 years). In all, 75% of the cohort had at least a 2-year follow-up (Fig. 2).

**Table 1:** Demographic and presurgical characteristics for the study cohort

Variables	IOMRI (*n* = 30)
Gender	
Male	20 (67)
Female	10 (33)
Age at onset^a^	5.0 ± 0.8 (0.1–14)
Age at surgery^a^	12.1 ± 1.0 (0.6–20.2)
Duration of epilepsy^a^	7.1 ± 0.8 (0.5–19.7)
Pre-op Phase II evaluation completed	24 (80)
Proximity/overlap of eloquent function	24 (80)
Motor	12 (40)
Speech	10 (33)
Vision	3 (10)
Concordance with Phase II localization	
PET (*n* = 18)^b^	15/19 (79)
SPECT (*n* = 11)^b^	8/11 (73)

^a^(years; mean ± SEM); range in parentheses

^b^The subset size that underwent the respective study

The majority (80%) of subjects underwent Phase II evaluation with SEEG electrode implantation (*n* = 24). Proximity and/or overlap between Phase II-derived EZ and eloquent function was observed in 80% of subjects (*n* = 24), most commonly for motor (40%, *n* = 12) and speech (33%, *n* = 10) function, respectively.

### Surgical resection of the hypothesized epileptogenic zone

Peri- and post-operative surgical variables are summarized in Table 2. Resections were guided by neurophysiological monitoring for motor function through a combination of cortical mapping, motor evoked potentials and subcortical mapping/stimulation in 47% (*n* = 14) of cases. Surgical resection of the hypothesized EZ was undertaken through a tailored, lesionectomy-plus resection in 50% (*n* = 15) of subjects, followed by a lobectomy in 47% (*n* = 14) of all subjects.

**Table 2:** Surgical and outcome variables for the study cohort

Variables	IOMRI application (*n* = 30)
**Surgical adjuncts utilized**	
ECOG	12 (40)
DTI-merged neuronavigation	15 (50)
**Neurophysiological monitoring**	
Cortical mapping	14 (47)
Motor evoked potentials and subcortical mapping	14 (47)
**Lobar localization for resection**	
Temporal	11 (37)
Frontal	11 (37)
Parietal	2 (6)
Insular	2 (6)
Multi lobar	4 (13)
**Type of surgical resection**	
Lesionectomy	1 (3)
Lesionectomy plus	15 (50)
Lobectomy	14 (47)
**Pathological diagnosis**	
FCD	10 (33)
Gliosis	9 (30)
Hippocampal sclerosis	5 (17)
Encephalocele	2 (6)
Others^b^	4 (13)
**IOMRI evaluation**	
Residual tissue identified on IOMRI study	22 (73)
Additional surgical resection undertaken	22 (73)
Total OR time^a^ (minutes)	544 ± 20
MRI time^a^ (minutes)	53 ± 3
Residual tissue identified on POD#1 MRI	0 (0)
**Morbidity/complications**	
Temporary unexpected post op deficit	5 (16)
Permanent unexpected post-operative deficit	1 (3)
**Seizure outcome**	
Engel I	24 (80)
Engel II	3 (10)
Engel I + II	27 (90)
Engel III	3 (10)
**Follow-up**^a^	4.2 ± 0.3 (range: 1.6–7.3)

^a^(mean ± SEM)

^b^*n* = 1 for heterotopia, polymicrogyria, non-diagnostic, and tuberous sclerosis, respectively

Lobar localization for the tailored lesionectomy plus resection sub group (*n* = 15), most commonly included 6 subjects (40%) with frontal and 3 subjects with temporal and parietal localization each (20%). Within the lobectomy sub group (*n* = 14), 9 subjects underwent a temporal lobectomy (64%) and 4 subjects underwent a frontal lobectomy (29%). One subject underwent a combined frontal–temporal lobectomy. Focal cortical dysplasia (33%, *n* = 10) and gliosis (30%, *n* = 9) were the most common pathological diagnoses followed by hippocampal sclerosis (17%, *n* = 5).

### Intraoperative electrocorticography

In all 12/30 (40%) of all subjects underwent iECOG monitoring during surgical resection (Table 2). The decision algorithm for incorporating iECOG was based on Phase II monitoring, in instances when wider irritative zones extending beyond the EZ were identified. Of these, 8/12 (67%) had identifiable baseline epileptiform discharges prior to resection. Within this subgroup of subjects (*n* = 8) with baseline epileptiform discharges prior to resection, repeat ECOG post resection showed resolution in 4/8 (50%) subjects. In 3/8 (37%) subjects with persistent epileptiform discharges following resection, additional tissue was removed and a subsequent repeat iECOG was negative. In 1 subject, epileptiform discharges overlapped language areas and further iECOG guided resection was not undertaken to avoid functional deficit. Five subjects (*n* = 5) with baseline iECOG abnormal activity had underlying FCD.

### IOMR imaging and impact on surgical resection

**Fig. 3:**
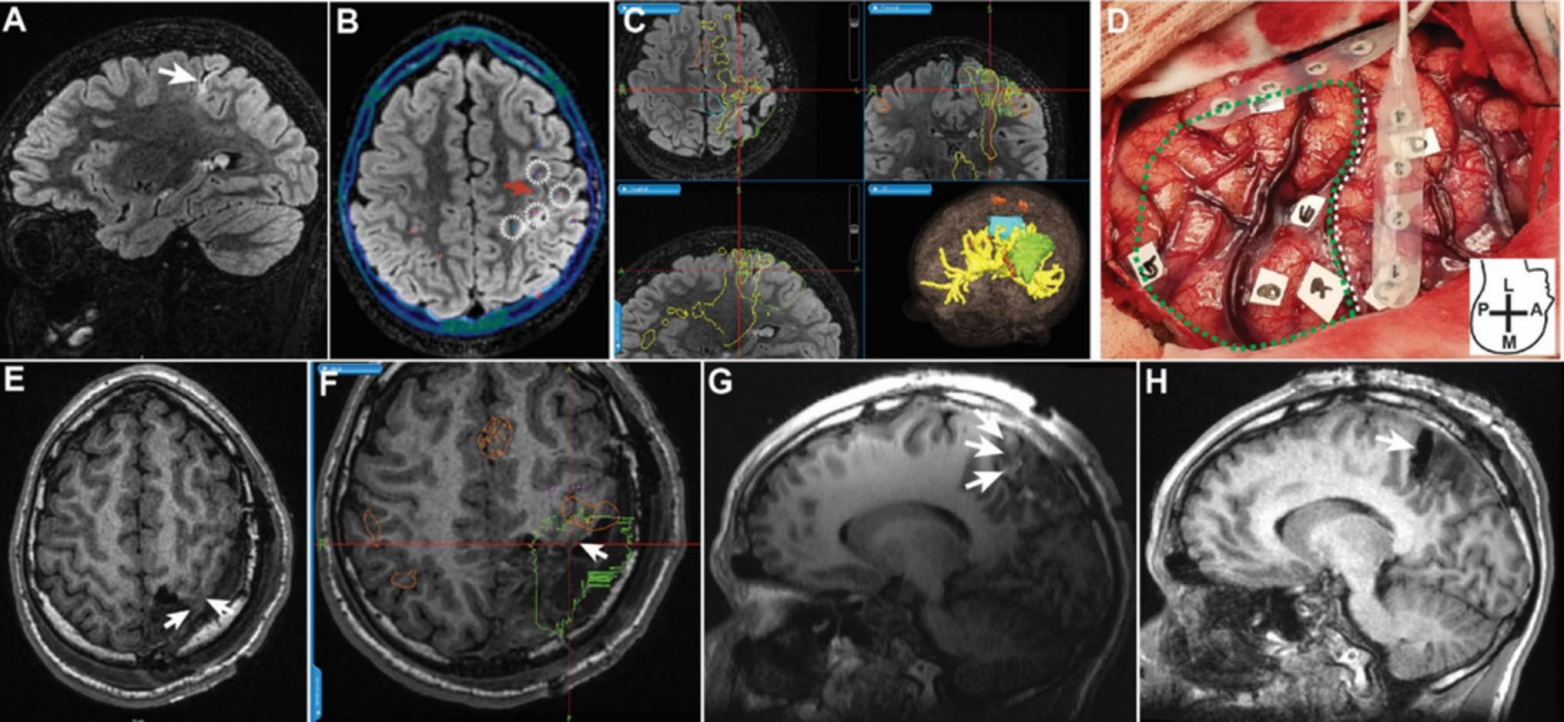
Illustrative example demonstrating residual, peri-lesional EZ on IOMRI study. A 13-year-old presented with right Rolandic epilepsy with right hemi body sensory-motor symptoms and radiographic evidence of gliosis within left post central gyrus (arrow, (**A**) sagittal FLAIR image). Stereo-EEG was undertaken with posterior frontal and parietal coverage. **B** Axial, FLAIR MRI and post SEEG-CT merged view indicating motor strip (red arrow) and selected peri-Rolandic SEEG trajectories (white, dashed circles). Epileptogenic zone (EZ) in anterior parietal region was delineated based on seizure onset and propagation patterns as evidenced on SEEG monitoring and results of stimulation and mapping. The EZ (green) was delineated in **C**, 3-D view on (Medtronic) neuronavigation software, showing adjacent leg motor area (blue) and corticospinal tract reconstruction (yellow). **D** Intraoperative image with surface labels corresponding to SEEG-defined boundaries of the EZ (green-dashed), central sulcus (white-dashed) and surface strip electrode for train-of-five MEPs. Anterior resection was undertaken till monopolar subcortical stimulation thresholds of 5–7 mA was encountered. Residual volume at anterior boundary of EZ (white arrow) was identified on **E** axial T1 weighted IOMRI image, and verified on comparison with EZ delineated on **F** navigation software. Resection was extended anteriorly till monopolar subcortical stimulation thresholds of 2–3 mA. Comparison of corresponding sagittal views on IOMRI **G** and POD#1 **H** images, white arrows, indicates complete EZ resection

**Fig. 4:**
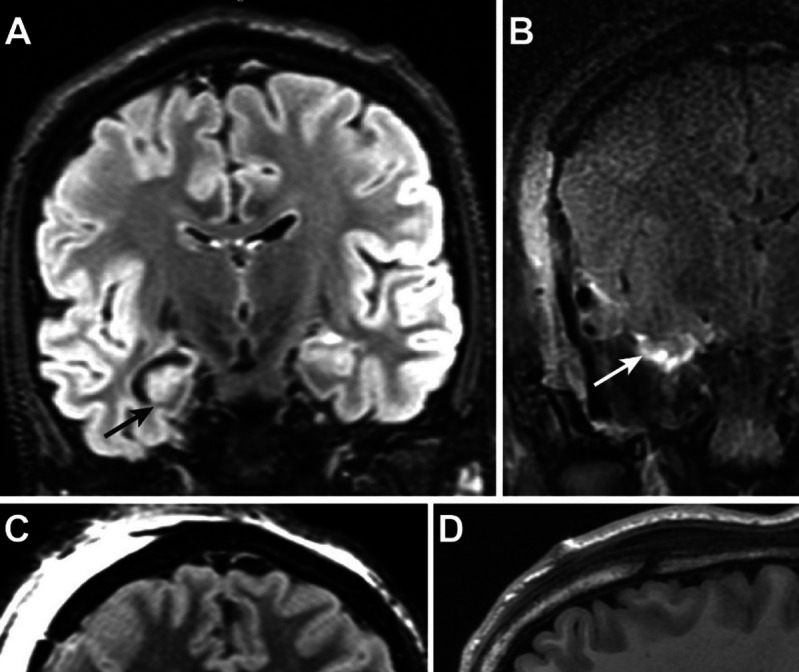
Illustrated example for residual EZ in temporal onset epilepsy. A 10-year with temporal onset seizures diagnosed through Phase I and II evaluation underwent a right anterior temporal lobectomy. **A** Pre-operative FLAIR images indicate mesial hippocampal sclerosis. **B** IOMRI corresponding image shows residual superior amygdala tissue (arrow), that was additionally resected. Post-operative, **C** FLAIR image shows complete resection, extending superiorly to the carotid-choroidal landmark (white – dashed line and white small arrows) on **D** T1 sagittal view

Mean time for IOMRI completion was 53 ± 3 min. In 22 subjects (*n* = 22/30; 72%), incomplete resection of the hypothesized EZ was identified on the IOMRI study (Table 3). Residual tissue was most often visible (64%) in the lesion perimetry (illustrative example, Fig. 3), adjoining insular cortex and within the subcallosal region. Incomplete resection was also noted within the superior aspect of amygdala and temporal piriform cortex (36%; illustrative example, Fig. 4). All 22 subjects with identifiable, residual hypothesized EZ on the IOMRI study, underwent additional tissue removal. No radiographic evidence of residual tissue was identified on POD 1 imaging for the 22 subjects that underwent IOMRI—guided repeat resection.

**Table 3:** Surgical and Outcome variables for cohort that underwent repeat IOMRI-guided resection (*n* = 22)

Variables	IOMRI application (*n* = 22)
**Lobar localization for resection**	
Temporal	8 (36)
Frontal	7 (32)
Parietal	3 (14)
Insular	2 (3)
Multi lobar	3 (14)
**Type of surgical resection**	
Lesionectomy plus	10 (45)
Lobectomy	12 (55)
**IOMRI evaluation**	
Additional surgical resection undertaken	22 (100)
Localization for additional IOMRI-guided resection	
EZ periphery	14 (64)
Amygdala and temporal stem	8 (36)
Residual tissue identified on POD#1 MRI	1 (3)

### Post-surgical seizure outcome and surgical morbidity

At last follow up, 80% (*n* = 24/30) of patients were seizure-free (Engel I) (Table 2). Seizure improvement (Engel I + II) outcomes were observed in 90% (*n* = 27/30) of the cohort. An Engel III outcome was observed in 3 subjects (10%). Table 4 summarizes the perioperative characteristics for 3 subjects that had unfavorable seizure control outcome (Engel III + IV) following surgery.

**Table 4:** Clinical summary of subjects with unfavorable seizure outcomes (Engel III + IV) following surgery

Subject ID	#1	#2	#3
Gender (M/F)	M	F	F
Age at onset (years)	0.5	10	0.3
Age at surgery (years)	9.1	17.8	2.6
Duration of epilepsy (years)	8.6	7.8	2.3
Phase II SEEG completed	Yes	Yes	yes
Area of resection	Right temporal	Right parietal	Right frontal
Eloquent cortex overlap	No	Yes	Yes
Eloquence function overlap	-	Motor	Motor
Surgical resection	Lobectomy	Lesionectomy plus	Lobectomy
Location of residual lesion on POD#1 MRI	-	-	Posterior frontal margin
Pathological diagnosis	Tuberous sclerosis	Gliosis	FCD
Follow up years	1.75	0.5	1.6
Temporary post op neurological deficit	-	hemiparesis	-
Permanent post op neurological deficits	-	-	-
Engel status at last follow up	III	III	III
Reason for surgical failure	Distant bilateral epileptogenic tubers	Motor function overlap	Motor function overlap

*TS *tuberous sclerosis, *FCD* focal cortical dysplasia, *HS *hippocampal sclerosis

Four subjects (13%) experienced temporary motor deficits with hemiparesis that resolved in 3 subjects by 3 months following surgery. One subject (*n* = 1/30; 3%) had residual spastic weakness in contralateral motor function, and was ambulatory with an ankle prosthesis.

## Discussion

Our results underscore the adjunct role of IOMRI in surgical treatment of DRE by enabling intraoperative confirmation of resection of the hypothesized EZ and eliminating the incidence of inadvertent incomplete resections. We observed a low incidence of surgical failure in our cohort, with follow-up to 4 years. In contrast to previous studies, our results over an extended follow-up period, elucidate long-term epilepsy outcomes for IOMRI-assisted surgery for DRE, that are associated with a low incidence of repeat resection to address epilepsy recurrence.

### Mitigation of early surgical failures

Surgical failures following epilepsy resections occur due to the following: (1) an inaccurate localization hypothesis, (2) residual EZ following surgery due to incomplete resection or functional constraints, (3) non-contiguous, secondary EZ(s), and finally (4) progression in underlying epilepsy with generation of de novo epileptic foci [19, 20]. Up to 50% of postoperative seizure recurrences tend to occur in the first 12 months following resection [4, 21]. Early surgical treatment failures are most consistently attributable to incomplete resection of the putative EZ [22–26]. Extended resection along initial surgical margins, directed at residual histopathological or electrographic abnormalities, is often associated with seizure relief during repeat resections [27].

In select epileptogenic lesions like bottom-of-sulcus dysplasias, electrophysiological and pathological evidence suggests that the radiographic lesion matches the zone of epileptogenicity [28], thereby suggesting that removal of peri-lesional tissue does not impact seizure outcomes [29]. For these epileptogenic lesions, a complete lesionectomy may be sufficient for removal of the EZ. However, in up to two-thirds of patients with epilepsy secondary to FCD, the EZ encompasses the lesion and adjacent peri-lesional cortex [30]. Chassoux et al. demonstrated that the EZ corresponded to the histological cortical dysplasia lesion in 82% of cases [31], thereby suggesting that in up to 20% of subjects, a lesionectomy approach is associated with a risk of incomplete EZ resection. In addition, epileptogenic lesions like dysplasias may be inaccurately delimited, despite 3-Tesla imaging [32]. Seizure outcomes following resection of most epileptogenic lesions therefore correlate with complete resection of the MR-visible lesion and peri-lesional cortex associated with epileptogenic potential or electrographic abnormalities [33]. These observations underscore the risk of incomplete EZ resection, if the radiographic lesion is used as the surrogate, radiographic metric for the EZ, and by extension, the intended surgical resection target.

Our study examined incomplete EZ resection as a modifiable, surgical risk factor associated with early surgical treatment failures. Our cohort consisted of non-tumoral epileptogenic lesions like FCD and gliosis, and is representative of neocortical epilepsy in pediatric subjects (Table 1). These epileptogenic lesions are often ill-defined, and are usually associated with EZ(s), that are larger than the radiographic lesion. We focused our study hypothesis on determining the impact of IOMRI on improving seizure outcomes, and mitigating early surgical failures, by confirming completeness of resection of the hypothesized EZ. The hypothesized EZ was presurgically identified on a volumetric basis, using Phase I- and Phase II-derived clinical, electrophysiological and radiographic data and included the MR-visible lesion and peri-lesional tissue associated with early ictal spread. We utilized anatomical boundaries with functional restrictions to refine the volumetric boundaries of the hypothesized EZ. In our cohort, only 1 subject underwent a lesionectomy while 97% (n = 29/30) of subjects either underwent a tailored, lesionectomy plus resection (50%) or a lobectomy (47%). We suggest that IOMRI can guide surgical resection effectively if the hypothesized, volumetric EZ is used as a radiographic metric to assess completeness of resection, instead of relying on (MRI-visible) lesional boundaries.

### Impact of IOMRI on seizure outcomes

Meta-analyses of studies evaluating the impact of IOMRI in epilepsy surgeries have demonstrated higher odds of achieving complete EZ resection (OR 3.19–4.75) and improving seizure outcomes (OR 3.80; 95% CI: 1.97–7.32) [34] [35]. Table 5 summarizes surgical and outcomes data for pediatric DRE subjects that underwent resection for non-tumoral epileptogenic lesions. However, several limitations were noted in these studies [35]. In a systematic review of 32 case series evaluating IOMRI-guided epilepsy resections [36], only 4 studies included subjects that underwent resection for epileptogenic lesions like cortical malformations, and had documented outcome measures of total resection [11, 37–39]. Only one study defined the methodological basis for identifying the EZ through a combination of the radiographic lesion and iECOG [38]. Notably, this cohort had limited follow-up of 5 months [37]. An a priori EZ was not defined in one study [39], and was based on lesionectomy-approaches in another study [11]. Previous reports also included mixed patient cohorts with both epileptogenic and non-epileptogenic lesions and surgeries involving resection and disconnection approaches [40, 41]. Some studies have not demonstrated change in reoperation rates [11] or seizure control outcomes [39] following IOMRI. Notably, seizure freedom in the cohort with ill-defined lesions was 50–57% in the study by Warsi et al. [39] and was attributed to inability to complete planned resection or an inaccurate localization hypothesis. These findings may reflect limitations of lesionectomy approaches, and heterogeneity in delineation of the EZ.

**Table 5:** Summary of previous publications focusing on IOMRI assisted resection of pediatric patients with non-tumoral epileptogenic lesions

Author year	No of pts	Mean age	Resection type	Histological diagnosis	Rate of IOMRI guided resection	Final rate of GTR	Engel status	Follow- up period for reported outcome
Eid et al.[11]	21	8.2 ± 1.6	lesionectomy	FCD – 12; HS – 6; TS – 3	9/12 – 75%	73%	76% Engel I + II	3.7 ± 1.9 years
Warsi et al. [39]	27	8.1 ± 5.5	Lesionectomy and lesionectomy plus	-	29% ^a^	NR	57% Engel I. 79% Engel I + II	2 years
Roessler et al. [8]	177	37.21(5 – 69)	Lesionectomy – 70%; Lesionectomy plus – 30%	FCD – 31; HS – 146	FCD – 12.9%; HS – 5.5%	87%	FCD - 50% Engel 1. HS - 79% Engel I	36 months (3 months – 10.8 years)
Sacino et al. ^b^ [37]	12	8.8 ± 1.6	Lesionectomy	FCD – 12	42%	100%	92% Engel 1	3.5 months ± 1.0 months
Sacino et al. ^b^ [42]	10	9.6 ± 2.3	Not specified	FCD – 10	NR	NR	90% Engel I	16.5 months
Sacino et al. ^b^ [10]	11	9.1 ± 2.0	NR	FCD – 11	36%	91%	82% Engel I	5.72 months (0.46–10.6)
Tejeda et al.[41]	45	9.3	Lesionectomy and mesial temporal resections	FCD – 22 HS – 3	27%	-	NR	NR
Kurzbuch et al. [40]	23	10 (1 – 18)	NR	FCD	63%	-	71% Engel I	1 year

^a^ 3 patients underwent repeat resections

^b^ Overlapping study period with common patients

Our surgical workflow utilized the hypothesized EZ as the intended surgical goal, with IOMRI assistance, to verify completeness of resection. Our results demonstrate that complete, MRI-verifiable resection of the hypothesized EZ, is associated with seizure outcomes of seizure freedom in 80% of subjects (Engel I) and epilepsy improvement in 90% of subjects (Engel I + II) with minimum 1.5 year follow-up. Unlike previous reports (Table 1) [34, 35], our mean follow-up period of 4.2 ± 0.3 years, with at least 75% of subjects with ≥ 2 year follow-up (Fig. 2), indicates that completeness of resection of the hypothesized EZ, with IOMRI assistance, is associated with sustainable seizure control outcomes [20, 37, 39]. We did not observe any surgical failures in subjects that underwent complete, verifiable resection of the hypothesized EZ. No subject in our series had residual EZ and none required reoperation following primary resection. We hypothesize that the risk of early surgical failure, commonly attributable to incomplete resection, can be reduced by application of IOMRI as a surgical adjunct during epilepsy surgery. Notably, for patients experiencing inadequate improvement in seizure control (Engel III status, *n* = 3, Table 4), seizure recurrence was attributed to incomplete resection of the hypothesized EZ, due to widespread epileptogenic network (*n* = 1; etiology tuberous sclerosis) and functional constraints (*n* = 2).

### IOMRI—assisted surgical resection

In our cohort, residual tissue was most commonly identified at the periphery of the hypothesized EZ, adjoining insular cortex, the subcallosal region, superior amygdala and temporal-piriform cortex respectively. Residual mesial-temporal tissue was identified through IOMRI in up to 50% of patients undergoing temporal lobectomy [43]. Incomplete resection of mesio-temporal structures and residual piriform cortex is a recognized risk factor for recurrent postoperative seizures for temporal onset epilepsy [44]. Our study experience suggests that accurate delineation and resection of superior amygdala and piriform cortex may be limited by dependence on intraoperative surgical landmarks. IOMRI-assisted temporal resections in our study, most commonly involved repeat resection of residual tissue within these surgical regions. An anterior temporal lobectomy was undertaken for temporal epileptogenic lesions since tailored- and lobar resections are more likely to be associated with seizure freedom than lesionectomies and sub-lobar resections [45]. Temporal resections in our series included the focal lesion (HS, FCD), and the lateral neocortex that may represent an irritative and/or early propagation zone [46].

The incidence of additional resection guided by IORMI assistance varies between 29 and 42% [34, 35]. Comparison of intraoperative and postoperative MRI studies in our series, indicates a 73% incidence of incomplete resection (*n* = 22) of the hypothesized EZ, based on intraoperative surgeon assessment. Our rate of repeat resection, as guided by IOMRI, was higher than previous reports, and may reflect a combination of the learning curve for incorporating IOMRI within the surgical work flow and the availability of IOMRI producing a bias towards an initial conservative resection, that can be extended further after IOMRI completion (Fig. 3). Notably, there was no radiographic evidence of residual tissue within the hypothesized EZ on post-operative day 1 MRI, with exception of subjects that underwent (intentional) limited resection due to functional constraints.

### Limitations

There are several limitations in this study. Our results should be interpreted in light of a lack of a control group, the retrospective nature of data collection and a single-center study cohort. The decision to undertake intraoperative imaging was not assigned randomly and the observational nature of our study precludes treatment recommendations. Usage bias exists since the predetermined IOMRI availability may limit initial resection till the IOMRI study is completed, leading to overutilization of intraoperative imaging. Seizure outcomes in DRE epilepsy are also influenced by accuracy of the localization hypothesis and variability in determination of surgical candidacy. The application of iECOG to guide EZ resection in a subset of DRE subjects may also confound study results. iECOG was used when wider irritative zones extending beyond the EZ were identified based on Phase II monitoring, consistent with a meta-analysis that showed clinical utility for iECOG in FCD due to frequent, well-localized epileptiform discharges, and characteristic inter-ictal activity patterns attributable to FCD [42].

The clinical pathway for diagnostic evaluation and surgical treatment for our cohort is standardized, and our results are therefore likely to be generalizable at other epilepsy centers. Despite increased operative and anesthetic time, incorporation of IOMRI is more cost-effective than pharmacological treatment of DRE [47]. Though repeat epilepsy surgery may improve seizure control, there is an increased risk of surgical morbidity with repeat resections [26].

## Conclusion

The sustained rate of seizure freedom in pediatric DRE subjects undergoing IOMRI-assisted surgery in our series, supports our hypothesis of undertaking a complete, verifiable resection of the hypothesized EZ in order to reduce the rate of early surgical failures. Since complete EZ resection is the strongest predictor for seizure freedom, we suggest that seizure outcomes observed in our cohort, over an extended follow up, are likely due to verifiable surgical resection of the entire hypothesized EZ, assisted by IOMRI. We did not observe any surgical failures for subjects that underwent complete, verifiable resection of the hypothesized EZ.

## Data Availability

No datasets were generated or analysed during the current study.
